# Exploring mechanisms of excess mortality with early fluid resuscitation: insights from the FEAST trial

**DOI:** 10.1186/1741-7015-11-68

**Published:** 2013-03-14

**Authors:** Kathryn Maitland, Elizabeth C George, Jennifer A Evans, Sarah Kiguli, Peter Olupot-Olupot, Samuel O Akech, Robert O Opoka, Charles Engoru, Richard Nyeko, George Mtove, Hugh Reyburn, Bernadette Brent, Julius Nteziyaremye, Ayub Mpoya, Natalie Prevatt, Cornelius M Dambisya, Daniel Semakula, Ahmed Ddungu, Vicent Okuuny, Ronald Wokulira, Molline Timbwa, Benedict Otii, Michael Levin, Jane Crawley, Abdel G Babiker, Diana M Gibb, for the FEAST trial group

**Affiliations:** 1 Wellcome Trust Centre for Clinical Tropical Medicine, Department of Paediatrics, Faculty of Medicine, St Marys Campus, Norfolk Place, Imperial College, London W2 1PG, UK; 2 Kilifi Clinical Trials Facility, KEMRI-Wellcome Trust Research Programme, PO Box 230, Kilifi, Kenya; 3 Medical Research Council (MRC) Clinical Trials Unit, Aviation House, 125 Kingsway London, WC2B 6NH, UK; 4 Department of Paediatrics University Hospital of Wales Heath Park, Cardiff, CF14 4XW, Wales, UK; 5 Department of Paediatrics, Mulago Hospital, PO Box 7070, Makerere University, Kampala, Uganda; 6 Department of Paediatrics, Mbale Regional Referral Hospital Pallisa Road Zone, PO Box 921, Mbale, Uganda; 7 Department of Paediatrics, Soroti Regional Referral Hospital, PO Box 289, Soroti, Uganda; 8 Department of Paediatrics, St Mary's Hospital, PO Box 180, Lacor, Uganda; 9 Department of Paediatrics Joint Malaria Programme, Teule Hospital, PO Box 81, Muheza, Tanzania; 10 Joint Malaria Programme, PO Box 2228, KCMC, Moshi, Tanzania

**Keywords:** Africa, children, clinical trial, fluid resuscitation, human albumin solution, mortality, saline, shock, terminal clinical events

## Abstract

**Background:**

Early rapid fluid resuscitation (boluses) in African children with severe febrile illnesses increases the 48-hour mortality by 3.3% compared with controls (no bolus). We explored the effect of boluses on 48-hour all-cause mortality by clinical presentation at enrolment, hemodynamic changes over the first hour, and on different modes of death, according to terminal clinical events. We hypothesize that boluses may cause excess deaths from neurological or respiratory events relating to fluid overload.

**Methods:**

Pre-defined presentation syndromes (PS; severe acidosis or severe shock, respiratory, neurological) and predominant terminal clinical events (cardiovascular collapse, respiratory, neurological) were described by randomized arm (bolus versus control) in 3,141 severely ill febrile children with shock enrolled in the Fluid Expansion as Supportive Therapy (FEAST) trial. Landmark analyses were used to compare early mortality in treatment groups, conditional on changes in shock and hypoxia parameters. Competing risks methods were used to estimate cumulative incidence curves and sub-hazard ratios to compare treatment groups in terms of terminal clinical events.

**Results:**

Of 2,396 out of 3,141 (76%) classifiable participants, 1,647 (69%) had a severe metabolic acidosis or severe shock PS, 625 (26%) had a respiratory PS and 976 (41%) had a neurological PS, either alone or in combination. Mortality was greatest among children fulfilling criteria for all three PS (28% bolus, 21% control) and lowest for lone respiratory (2% bolus, 5% control) or neurological (3% bolus, 0% control) presentations. Excess mortality in bolus arms versus control was apparent for all three PS, including all their component features. By one hour, shock had resolved (responders) more frequently in bolus versus control groups (43% versus 32%, *P *<0.001), but excess mortality with boluses was evident in responders (relative risk 1.98, 95% confidence interval 0.94 to 4.17, *P *= 0.06) and 'non-responders' (relative risk 1.67, 95% confidence interval 1.23 to 2.28, *P *= 0.001), with no evidence of heterogeneity (*P *= 0.68). The major difference between bolus and control arms was the higher proportion of cardiogenic or shock terminal clinical events in bolus arms (n = 123; 4.6% versus 2.6%, *P *= 0.008) rather than respiratory (n = 61; 2.2% versus 1.3%, *P *= 0.09) or neurological (n = 63, 2.1% versus 1.8%, *P *= 0.6) terminal clinical events.

**Conclusions:**

Excess mortality from boluses occurred in all subgroups of children. Contrary to expectation, cardiovascular collapse rather than fluid overload appeared to contribute most to excess deaths with rapid fluid resuscitation. These results should prompt a re-evaluation of evidence on fluid resuscitation for shock and a re-appraisal of the rate, composition and volume of resuscitation fluids.

**Trial registration:**

ISRCTN69856593

## Background

In the Fluid Expansion as Supportive Therapy (FEAST) trial, African children with shock randomized to early rapid fluid resuscitation (20 to 40 ml/kg boluses) with normal saline or 5% human albumin had a 3.3% increased absolute risk of death by 48 hours compared with no-bolus controls [1]. Following publication, commentary papers, letters and discussion groups have speculated on reasons for this surprising result [2-7], given that bolus resuscitation is the gold standard for shock-management in well-resourced countries (albeit based on weak levels of evidence) [8].

FEAST was a pragmatic trial conducted in African hospitals without ventilation facilities. It included children with shock caused by a heterogeneous group of conditions including sepsis [9] and malaria [10] but excluded those with gastroenteritis and severe malnutrition [11]. Consistency of adverse outcome was shown over all sites and in all possible subgroups, with no benefit of boluses observed for any working diagnosis [1], for any definition of shock [7,12-14], or presence or absence of anemia or under-nutrition. As reasons for harm caused by boluses remained unclear, we undertook further analyses to explore possible mechanisms and modes of death in children randomized to bolus resuscitation versus control. We hypothesized that boluses may cause excess deaths from neurological or respiratory events, particularly those relating to fluid overload. We explored the effect of boluses on 48-hour mortality according to signs and symptoms at enrolment (presentation syndrome, PS); predominant clinical syndrome in each patient prior to death (terminal clinical event, TCE); and changes in vital signs, measured prospectively at pre-specified times.

## Methods

### Trial design and population

The methods and the outcome of the trial have been reported in detail [1]. In brief, children, aged 60 days to 12 years, with severe febrile illness (classed as impaired consciousness (prostration or coma) and/or respiratory distress (increased work of breathing) plus clinical evidence of impaired perfusion (one of capillary refill time >2 seconds, lower limb temperature gradient, weak radial pulse volume or severe tachycardia) at six centers in Kenya, Tanzania and Uganda were enrolled into two strata according to systolic blood pressure [1]. Stratum A included 3,141 children without severe hypotension who were randomized to immediate bolus of 20 ml/kg (increased to 40 ml/kg after protocol amendment [1]) of 5% albumin (albumin-bolus: 1,050 children) or 0.9% saline (saline-bolus: 1,047 children), or no-bolus (control, maintenance fluids 4 ml/kg/hour: 1,044 children). The saline-bolus and albumin-bolus arms, but not the control arm, received an additional 20 ml/kg bolus at one hour if impaired perfusion persisted. In all three arms, further 40 ml/kg boluses of study fluid (saline for the control arm) were only prescribed beyond one hour if severe hypotension developed (see definition below). Stratum B included 29 children with FEAST entry criteria plus severe hypotension (defined as systolic blood pressure <50 mmHg if <12 months; <60 mmHg if 1 to 5 years; <70 mmHg if > 5 years) who were randomized to albumin or saline boluses of 40 to 60 ml/kg only. The primary endpoint was 48-hour mortality. Children with severe malnutrition, gastroenteritis, trauma, surgery or burns were excluded.

### Baseline and follow-up data collection

Trial clinicians completed a structured clinical case report form at admission. A venous blood sample was taken for immediate biochemical analyses with a handheld blood analyzer (iSTAT, Abbott Laboratories, Abbott Park, IL, USA), hemoglobin was measured with HemOcue (Ängelholm, Sweden), glucose and lactate were measured with an On-Call glucometer and a Lactate Pro meter respectively and HIV antibody testing. Blood smears for malaria parasites were prepared for immediate reading and subsequent quality control. Standardized clinical reviews were conducted at 1, 4, 8, 24 and 48 hours including vital signs and hemodynamic monitoring. A working clinical diagnosis was recorded at 48 hours as well as history of prior neurodevelopmental progress and any pre-admission neurological deficits. Children were managed on general pediatric wards; mechanical ventilation (other than short-term 'bag-and-mask' support) was not available. Basic infrastructural support for emergency care as well as oxygen saturation and automatic blood pressure monitors were provided for each site. All trial patients received intravenous antibiotics, antimalarial drugs (for those with falciparum malaria) and intravenous maintenance fluids (2.5 to 4 ml/kg/hour as per national guidelines) until the child was able to retain oral fluids. Antipyretics, anticonvulsants and treatment for hypoglycemia (blood sugar <2.5 mmol/l) were administered according to nationally agreed protocols. Children with a hemoglobin level <5 g/dl were transfused with 20 ml/kg of whole blood over 4 hours.

### Assignment of presentation syndrome and terminal clinical event

Prior to and throughout the trial, clinical staff received onsite training in triage and emergency life support management to optimize case recognition, implement supportive management and ensure protocol adherence. Throughout the hospital admission, severe adverse events were reported immediately and clinical features of pulmonary edema and raised intracranial pressure, and evidence of hypovolemia and allergic events were actively solicited. An independent clinician removed all references to randomized arm or fluid management prior to review by the Endpoint Review Committee (ERC), which included an independent chair (JE), five independent pediatricians (experienced in high dependency care and/or working in Africa), and center principal investigators. The ERC had access to 'blinded' clinical narratives, bedside vital observations (below), iSTAT results (baseline, 24 hours), microbiology, malaria and HIV status, and concomitant treatments. They adjudicated (blind to randomized arm) on whether fatal and non-fatal events could be related to bolus interventions and the main causes of death [1]. In addition, the ERC chairperson and another non-center ERC member reviewed all deaths occurring within 48 hours; using pre-specified criteria, they stratified all children by presentation syndrome (PS) and classified the predominant clinical mode of death (TCE).

### Presentation syndromes definitions

***Severe shock or acidosis presentation ***was any one of blood lactate >5 mmol/l [8], base excess >-8 mmol/l [10]; World Health Organization shock definition (all of cold hands or feet; capillary refill time >3 seconds; weak and fast pulse) [14] or moderate hypotension (systolic blood pressure (SBP) 50 to 75 mmHg in children aged <12 months, 60 to 75 mmHg in children aged 1 to 5 years, and 70 to 85 mmHg in children aged >5 years).

#### Respiratory presentation

hypoxia (oxygen saturation <92% measured by pulse oximetry) [15] plus one of history of cough, chest indrawing or crepitations [16].

#### Neurological presentation

coma or seizures at or immediately preceding hospital admission [16].

### Terminal clinical event syndrome definitions

#### Cardiogenic/cardiovascular collapse

signs of shock at the point of demise - severe tachycardia or bradycardia plus one of prolonged capillary refill time >2 seconds, cold peripheries or low SBP. If hypoxia was also present then this mode of TCE was a consensus view among ERC members, that circulatory failure was deemed to be the primary problem.

#### Respiratory

Ongoing or development of hypoxia (PaO_2 _<90%) with chest signs (crepitations or indrawing). Primary cause of death assigned as pneumonia and/or involving possible pulmonary edema.

#### Neurological

Possible raised intracranial pressure (high SBP or relative bradycardia) or, in children with severely reduced conscious level (Blantyre Coma Score ≤2 [17]), focal neurological signs, abnormal pupil response to light or posturing at the point of demise.

Children whose deaths were unwitnessed were assigned 'unknown' TCE.

### Analysis

The analysis focused on children in stratum A, in which 86% of deaths occurred within 48 hours. Stratum B (mortality 62% (18 out of 29)) was not included because all children received boluses [1]. Albumin- and saline-bolus arms were combined, as mortality was very similar in both. All comparisons between combined bolus and control arms were performed according to intention-to-treat, and all statistical tests were two-sided.

PS prevalence was described by randomized arm and 48-hour mortality was compared by randomized arm within each PS. Forest plots were constructed to show comparisons between arms for 48-hour mortality according to PS and all individual features of each PS. Hazard ratios for the comparison between bolus and no-bolus arms were estimated for different levels of oxygen saturation and hemoglobin from Cox proportional hazard models, with a single indicator for treatment group, the level of the parameter and an interaction between treatment group and parameter. Competing risks methods were used to estimate cumulative incidence curves and sub-hazard ratios to compare the two treatment groups in terms of TCEs [18].

Oxygen saturation, axillary temperature, heart rate, respiratory rate, SBP, glucose values and a composite measure of shock or impaired perfusion (defined as any of the following: capillary refill time >2 seconds, lower limb temperature gradient, weak radial pulse volume or severe tachycardia (<12 months, >180 bpm; 1 to 5 years, >160 bpm; >5 years, >140 bpm)) were summarized for survivors over time (at baseline, 1, 4, 8, 24 and 48 hours) with box and whisker plots and bar charts. Treatment groups were compared in terms of mortality after one hour, conditional on changes in shock and hypoxia parameters 1-hour post-randomization. Our analyses focused only on early changes where the number of deaths was similar across randomized arms; thereafter, because of excess deaths in bolus arms, results would be subject to survivorship bias.

### Ethics statement

Ethics Committees of Imperial College London, Makerere University Uganda, Medical Research Institute, Kenya and National Medical Research Institute, Tanzania approved the protocol.

## Results

**Figure 1:**
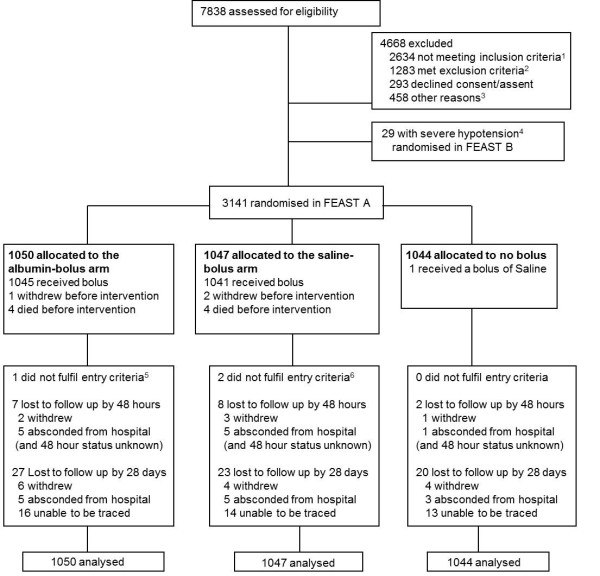
**Patient flow**. ^1^**Inclusion criteria**: Children aged >60 days and <12 years with severe febrile illness including impaired consciousness (prostration or coma) and/or respiratory distress (increased work of breathing) were screened for clinical evidence of impaired perfusion (shock) to be eligible for the trial. Impaired perfusion was defined as any one of the following: CRT 3 or more secs, lower limb temperature gradient, a weak radial pulse volume or severe tachycardia: (<12 months: >180 beats per minute (bpm); 12 months to 5 years: >160bpm; >5 years: >140 bpm). ^2^**Exclusion criteria**: Evidence of severe acute malnutrition (visible severe wasting or kwashiorkor); gastroenteritis; chronic renal failure, pulmonary edema or other conditions in which volume expansion is contraindicated; non-infectious causes of severe illness (68); if they already received an isotonic volume resuscitation. ^3^Other reasons for exclusion: child unable to return for follow-up (111), enrolled in a different study (65), no trial packs/fluid or blood (47), previously enrolled to FEAST (17), died (11), other (181), missing reason (26). ^4^Severe hypotension defined as systolic blood pressure <50mmHg if <12m; <60mmHg if 1-5years; <70mmHg if >5years- eligible children with severe hypotension were enrolled into FEAST B (see text) ^5^Child was not febrile (had no fever or history of fever). ^6^One child had severe hypotension and one child did not have impaired perfusion.

In FEAST stratum A, 3,141 children were randomized between 13 January 2009 and 13 January 2011 (1,050 albumin-bolus, 1,047 saline-bolus, 1,044 control) (Figure 1). Baseline characteristics were similar across arms and median age was 24 months (interquartile range 13 to 38 months). Overall, 2,398 (76%) had impaired consciousness (including 457 (15%) with unarousable coma), 1,172 (37%) had convulsions and 2,585 (83%) had respiratory distress. *Plasmodium falciparum *malaria parasitemia was present in 1,793 out of 3,123 (57%); severe anemia (hemoglobin <5 g/dl) in 987 out of 3,054 (32%); 1,070 out of 2,079 (52%) had a base deficit > 8mmol/L; 1,159 out of 2,981 (39%) had a lactate level >5mmol/l; 126 out of 1,070 (12%) had bacteremia (positive blood culture); and 10 out of 292 (3%) had meningitis (positive cerebrospinal fluid culture).

### Presentation syndromes

**Figure 2:**
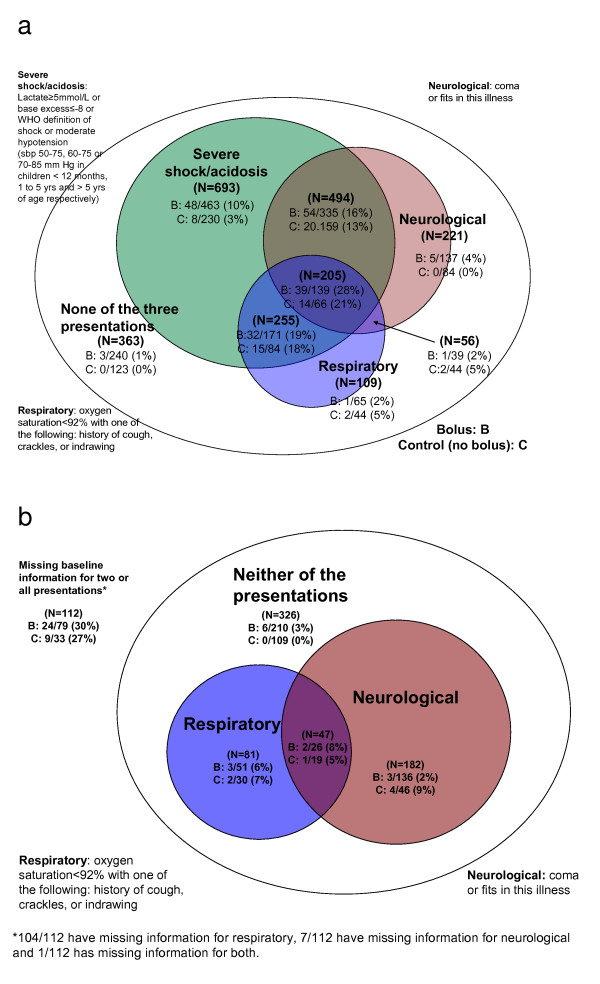
**Mortality at 48 hours by presentation syndrome**. **(a) **Complete information; n = 2,396. **(b) **Incomplete information; n = 745. 48-hour mortality by presentation syndrome and in bolus (albumin and saline) and control (no bolus) arms for those for which severe shock or acidosis (n = 633), or respiratory syndrome (n = 105) or neurological syndrome (n = 8) could not be ascertained. Areas are proportional to the size of subgroups. B: bolus arm; C: control arm.

Of the 3,141 children in stratum A, 2,396 (76%) could be classified into a single PS or a combination; 633 (20%) cases had missing base excess (589) or lactate (32) or blood pressure (12), thus precluding classification to shock or acidosis PS, but half of these had additional respiratory and/or neurological presentations (Figure 2a,b; Table S1 in Additional file 1). In 112 children (4%), information was missing on two or more PS. Of the 2,396 children with full information, 1,647 (69%) had severe metabolic acidosis or severe shock, 625 (26%) had respiratory presentations and 976 (41%) had neurological presentations, alone or in combination. The distribution of PS was balanced across randomized arms (Table S1 in Additional file 1).

#### Mortality by presenting syndrome

Mortality was greatest among children fulfilling criteria for all three PS (28% bolus, 21% control) and combined shock or acidosis and respiratory presentations (19% bolus, 18% control). The greatest differences in mortality between bolus and control groups was among those with all three PS (n = 205) and those with severe shock or acidosis PS alone (n = 698; 10% bolus, 3% control). These two groups represented 37% (898 out of 2,396) of classifiable cases. Mortality was lowest for respiratory presentation alone (2% bolus, 5% control) or neurological presentation alone (3% bolus, 0% control) (Figure 2a). A small number, 363 out of 2,396 (15%), had only FEAST entry criteria; three children died in this group (2% bolus; 0% control).

**Figure 3:**
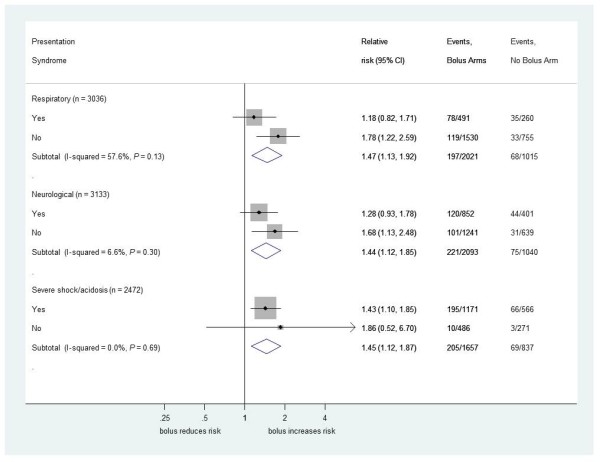
**Mortality risk at 48 hours for bolus compared to no bolus by presentation syndromes at baseline**. Forest plots comparing effect of bolus versus no bolus for each baseline presentation syndrome (respiratory, neurological or severe shock or acidosis); children could be assigned to more than one syndrome.

**Figure 4:**
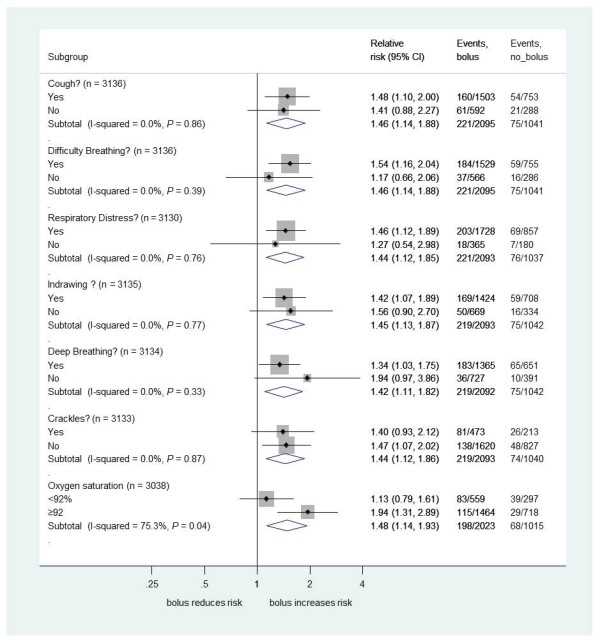
**Mortality risk at 48 hours for bolus compared to no bolus by individual respiratory symptoms/signs at baseline**.

**Figure 5:**
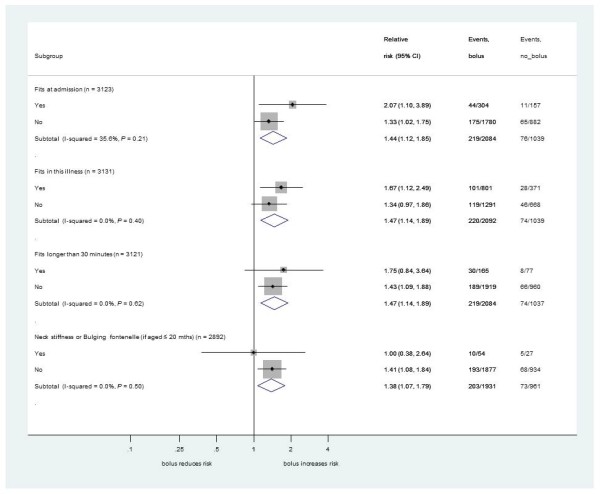
**Mortality risk at 48 hours for bolus compared to no bolus by individual neurological signs/symptoms at baseline**.

**Figure 6:**
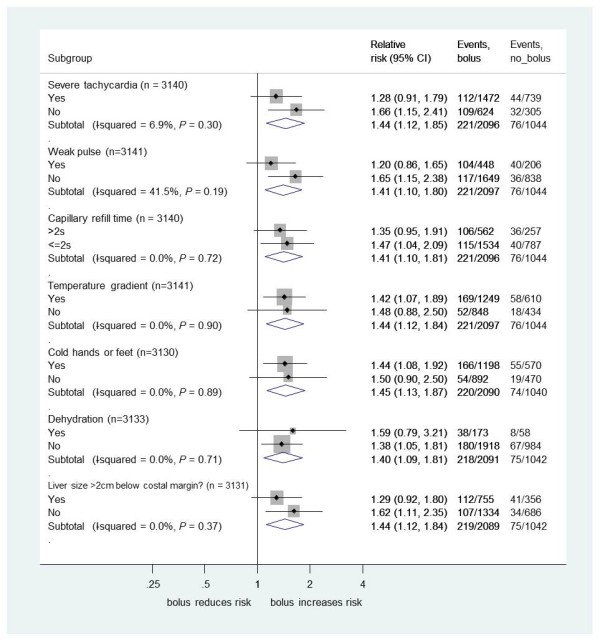
**Mortality risk 48 hours for bolus compared to no bolus by shock-related or systemic signs at baseline**.

We found no evidence that the excess 48-hour mortality in bolus arms versus the control arm differed by PS (Figure 3) or by individual clinical components of each PS (Figures 4, 5 and 6) (all *P*-values for heterogeneity ≥0.2). The exception was hypoxia (oxygen saturations <92%), present in 856 children (27%) at admission. As expected, hypoxia was a strong predictor of higher subsequent mortality, but there was statistically significant evidence that the excess mortality with boluses was greater in the subgroup without hypoxia at presentation (bolus versus control, relative risk (RR) 1.94, 95% confidence interval (CI) 1.31 to 2.89) than in the subgroup with hypoxia (RR 1.13, 95%CI 0.79 to 1.16; heterogeneity *P *= 0.04, Figure 4). This is also demonstrated with oxygen saturation as a continuous variable in Figure S2 in Additional file 1. Conversely, and as reported previously, the degree of anemia at admission had no significant impact on the effect of boluses on overall mortality [1,7], excess harm being evident in the bolus arms versus control across the whole range of baseline hemoglobin values (Figure S3 in Additional file 1).

### Terminal clinical events

In stratum A, 345 out of 3,141 children (11%) died; of these, 297 deaths (86%) occurred within 48 hours. Primary working diagnoses, recorded by clinicians and reported previously [1], included malaria 142 (48%), pneumonia or respiratory etiology 41 (14%); septicemia 27 (9%), anemia 27 (9%), meningitis 15 (5%), encephalitis 7 (2%), other diagnosis 12 (4%) and insufficient information 26 (9%). The ERC adjudicated 265 single and 32 (11%) combined TCEs: 247 (83%) were judged to have a primary cardiogenic, respiratory or neurological TCE [1].

**Figure 7:**
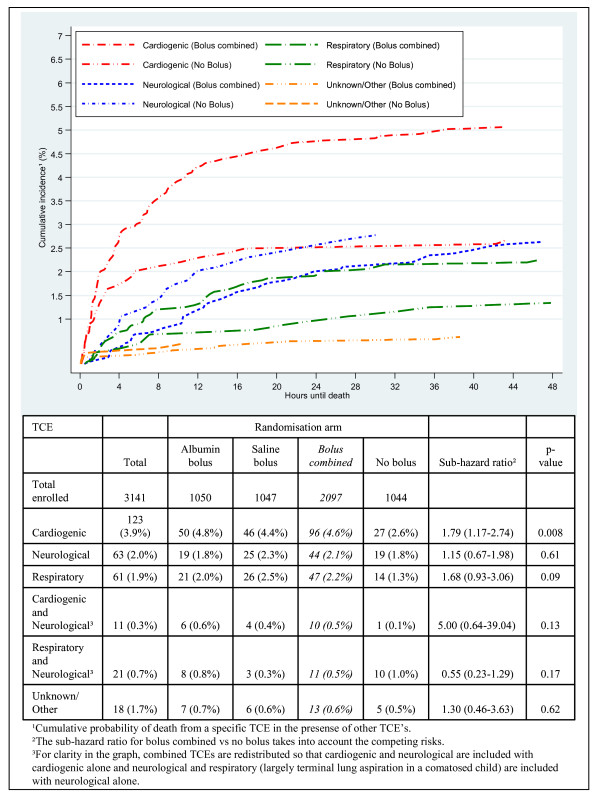
**Cumulative incidence of mortality for bolus combined and no bolus arms by terminal clinical events for 297 children who died within 48 hours**.

A cardiovascular or shock TCE was the most frequent overall (n = 123 (41%)); neurological and respiratory TCEs occurred in 63 children (21%) and 61 children (20.5%) respectively (Figure 7); in 18 children the TCE was unknown. As expected, TCE generally aligned with PS (Table S2 in Additional file 1).

#### Terminal clinical event-specific mortality by randomization arm

The major difference between bolus and control arms was the higher proportion of deaths adjudicated as having a cardiogenic or shock TCE in bolus arms, 96 (4.6%) compared with 27 (2.6%) in the control arm (sub-hazard ratio 1.79, 95%CI 1.17 to 2.74, *P *= 0.008, Figure 7). This difference was even greater when 39 modes of death in 39 children who died in the first hour (when bolus administration was incomplete) were excluded (79 (3.8%) compared with 19 (1.8%) respectively of modes of death were cardiogenic (sub-hazard ratio 2.09, 95%CI 1.27 to 3.45, *P *= 0.004)). Of note, and as expected, 25 out of 39 early deaths (64%) were cardiogenic.

We found no evidence for increased risk of neurological events (putative 'cerebral edema') with boluses: there were 44 neurological TCEs in bolus arms (2.1%) versus 19 (1.8%) in the control arm (*P *= 0.6). Respiratory TCEs (putative 'pulmonary edema') were marginally more common in bolus arms: 47 (2.2%) versus 14 (1.3%); *P *= 0.09 (Figure 7). No significant differences were found between albumin or saline boluses for any TCE.

The cumulative incidence of death by TCE for all children by bolus versus control arms is shown in Figure 7, where, for clarity, single and combined TCEs are redistributed so that cardiogenic and neurological TCEs are included with cardiogenic alone, and neurological and respiratory (largely terminal lung aspiration in a comatose child) are included with neurological alone. Cumulative incidence for individual and combined TCE categories is shown in Figure S4a,b in Additional file 1.

#### Terminal clinical events according to bolus volume, malaria status and hemoglobin

The effect of a bolus had similar patterns on TCEs in children receiving 20 ml/kg and 40 ml/kg (that is, before and after the protocol amendment), among children with and without malaria, and in those with and without severe anemia. In all groups, cardiogenic TCEs accounted for the greatest excess in mortality in the bolus versus control groups, with no evidence of heterogeneity (all *P*-values >0.1) (Tables S3a,b,c in Additional file 1).

#### Changes in hemodynamics, vital status and laboratory parameters over time

Box and whisker plots of individual bedside vital status observations, including heart rate, respiratory rate oxygen saturation, consciousness level and hypoglycemia (blood glucose <3 mmol/L) showed improvement over time, with few differences between bolus and control arms (Figure S1 in Additional file 1). The exception was the composite measure of impaired perfusion (first box and whiskers plot in panel in Figure S1 in Additional file 1), which by one hour had resolved more frequently in the bolus than control arms; 43% of bolus-recipients had no sign of impaired perfusion compared with only 32% in the control arm *(P ≤*0.001).

#### Hemodynamic responses and changes in oxygen status at one hour

The mortality at 48 hours was significantly higher among 1,881 children with persistent impaired perfusion (see analysis section for definition) at one hour (non-responders) compared with 1,198 responders (shock-resolution) (10% versus 4%, *P *<0.001, Table S4a in Additional file 1). However, despite greater improvements in perfusion in the bolus arm at one hour, excess mortality in bolus versus control arms was evident in non-responders (RR 1.67, 95%CI 1.23 to 2.28, *P *= 0.001) as well as responders (RR 1.98, 95%CI 0.94 to 4.17, *P *= 0.06), with no evidence that these were different (heterogeneity *P *= 0.68, Table S4a in Additional file 1).

Children with baseline hypoxia who remained hypoxic at one hour had increased risk of subsequent mortality compared with those whose hypoxia resolved (18% versus 7%, *P *<0.001, Table S4b in Additional file 1). There was no evidence to indicate that boluses were associated with increased mortality in the children with persistent hypoxia compared with control children (RR 0.71, 95%CI 0.43 to 1.18). Among children whose hypoxia had resolved by one hour, the RR of mortality for bolus versus control was 1.45 (95%CI 0.73 to 2.85, heterogeneity *P *= 0.1, Table S4b in Additional file 1).

A total of 175 out of 2144 children without hypoxia at baseline (8%) developed hypoxia by one hour and, as expected, had higher mortality compared with those who did not develop hypoxia (15% versus 5%, bottom panel of Table S4b in Additional file 1). Slightly more children in the bolus arms than in the control arm (129 (9%) versus 46 (7%)) developed hypoxia by one hour. However, excess risk of death in the bolus versus control arms was observed among both children who developed hypoxia (RR 1.96, 95%CI 0.71 to 5.39) and those who remained non-hypoxic (RR 2.64, 95%CI 1.53 to 4.54). Thus, overall, there was no evidence that development of *de novo *hypoxia by one hour impacted on the excess mortality in fluid bolus versus control arms (*P*-value for heterogeneity = 0.63, Table S4b in Additional file 1).

## Discussion

In this paper, we have explored possible mechanisms for the excess death rate among children randomized to receive rapid boluses of 20 to 40 ml/kg of 5% albumin or 0.9% saline fluid resuscitation compared with no-bolus controls. We found no evidence that excess 48-hour mortality associated with boluses differed by type of PS, by individual constituent components of each syndrome, or by baseline hemoglobin level. Remarkably, in every subgroup we examined, there was consistent evidence of harm by boluses. Paradoxically, the syndromes where most concern has been expressed over trial inclusion (respiratory or neurological alone and/or less severe shock criteria) not only had lower mortality overall, but also tended to have smaller differences between bolus and control, although care must be taken with interpretation of smaller subgroups. The only exception was hypoxia, present in a quarter of children at admission, which surprisingly appeared to be associated with significantly less harm from boluses. There appears to be no good rationale for this finding, which could have occurred by chance.

Even though this trial was conducted in settings with limited resources and no access to intensive care, the conduct of the trial complied to the highest standards of good clinical practice including adherence to intervention strategy and completeness of follow-up, 100% source document monitoring and the robust and blinded methodology for determining PS and TCE. An intention-to-treat analysis with no need for imputation for missing data minimized the likelihood of bias and underpinned the magnitude and importance of the unexpected findings of the trial and further analyses.

Noteworthy is that, consistent with global clinical experience, we observed a superior resolution of impaired perfusion by one hour in the bolus arms compared with the control arm. However, importantly, this did not translate into a superior outcome when compared with children with continued impaired perfusion: in both cases, boluses resulted in higher mortality. Mortality excess with boluses among children with and without hypoxia at baseline occurred to a similar extent irrespective of oxygen saturation status at one hour. Although *de novo *development of hypoxia at one hour was more common in the bolus arms, it was not associated with a significant increase in 48-hour mortality - suggesting that, if fluid boluses were causing pulmonary edema (and hypoxia), this was not a unifying mechanism for increased mortality from boluses. Moreover, there was little evidence that fluid overload was the mechanism for excess deaths with boluses from our analyses of neurological or respiratory TCEs. Overall, cardiovascular collapse was the main TCE and contributed most substantially to the excess mortality in the bolus arms compared to control, peaking at 2 to 11 hours post-bolus. Whilst it is possible that subtle effects of fluid overload on the lungs or brain could have been missed, our findings do not lend support to this hypothesis, particularly as the ERC review process was blind to randomization and used pre-specified TCE definitions.

A limitation of our trial was that we were unable to undertake invasive or point-of-care continuous monitoring to provide greater insight into TCEs, as in high-income intensive care settings. However, the availability of patient-centered variables from our bedside observations and laboratory data from most children at baseline has enabled further characterization of the trial participants into clinically relevant presentations (PS) in the context of where the trial was conducted. It has provided a more in-depth understanding of the spectrum of clinical groupings of children enrolled in the trial and the degree of adverse outcome fluid boluses had in these groups. Our bedside observations at pre-defined times following randomization, as well as adjudication of cause of death by the ERC, blind to randomized arm and using pre-specified criteria (TCE), provide more speculative data but remain informative because they are largely operator-independent. These methodologies are central to the internal validity of our analysis, which sought to minimize systematic bias.

Whereas initial improvement in circulatory status following bolus resuscitation is consistent with global clinical experience [8,12,19], the observation of excess subsequent cardiovascular collapse and excess mortality, even in the early responders, was only made possible because of exemplary adherence to randomized allocation, and in a trial protocol in which further boluses were given to only (very few) children developing severe hypotension [12]. The possibility that fluid resuscitation lead to hemodilution [20,21] in already anemic children, reducing oxygen delivery to the myocardium [22] and leading to ischemia and cardiac dysfunction, is largely ruled out by the lack of heterogeneity in the TCEs by hemoglobin level, and by the analysis showing excess harm with fluids across the whole range of hemoglobin values. These findings challenge the presumption that early and rapid reversal of shock by fluid resuscitation translates into longer-term survival benefits [8,23,24] in settings where intensive care facilities are not available. They do raise a possibility that rapid fluid resuscitation may cause adverse effects on vascular hemodynamics and myocardial performance, driving the requirement for inotropic and pressor support. Rapid restoration of microcirculatory perfusion may come at the expense of requiring other components of the sepsis bundle, including inotropes and ventilation. This possibility could be explored in future clinical and preclinical research examining the natural history of shock in patients managed by maintenance only (as in the control arm) and with fluid boluses.

Adverse effects of hyperchloremia, at the doses given in this trial, remain controversial [25-29]. Intriguingly, the most deleterious effects of boluses were in those patients with severe acidosis at baseline, the group with the least *a priori *equipoise regarding the potential benefits of fluid resuscitation - lending support to the notion of adverse effects of the resuscitation fluids on acid-base equilibrium. Alternatively, this may indicate lethal reperfusion injury [30] or a surge of cytokines [31] in cases with advanced shock. As previously suggested [1], shock may be an adaptive, time-dependent response sustaining children through a prolonged period prior to hospital admission - only to die within hours of reperfusion.

Whatever the explanation, these findings raise important questions about the pathophysiological mechanisms of shock and goal-directed management, questioning whether the protocol-driven requirement for inotropes and vasoactive drugs has been driven, in part, by aggressive fluid challenge [12]. The 2008 Surviving Sepsis Campaign Guidelines, informed by a modified Delphi process, graded the pediatric recommendation (20 ml/kg boluses over 5 to 10 minutes up to 60 ml/kg) as 2C, indicating a weak recommendation with low quality of evidence. The pediatric studies on which these recommendations were based included only two retrospective, observational studies of initial resuscitation volume on the outcome of children with putative sepsis from a single tertiary referral center, involving 34 and 91 children respectively [19,24]. The inclusion criteria for both studies were children who survived to intensive care unit admission, but were inotrope-dependent and had pulmonary arterial catheters *in situ*. Higher initial fluid boluses and early shock reversal in 9 and 24 children in the two respective studies were associated with improved global outcome. However, the study design and the patient population had major limitations in terms of survivorship bias and external validity to other settings. Other sources of evidence that were referenced to inform fluid resuscitation guidelines include dengue shock. We suggest these are largely irrelevant to the management of sepsis, because shock as a complication of dengue occurs 7 to 10 days after fever defervescence, secondary to gross intravascular leakage leading to vomiting, abdominal pain, increasingly tender hepatomegaly, narrow pulse pressure and hemoconcentration [32]. The American College of Critical Care Medicine guideline, and similar guidelines, nevertheless have been adopted by many countries worldwide where point-of-care testing and intensive care facilities are available and are considered to be best practice. These are now being recommended as standards of care for resource-poor settings [33,34]; indicating that the FEAST trial had limited generalizability because the adverse effects of fluid boluses were largely confined to children with malaria and/or anemia [33]. The data presented in the original manuscript [1], subsequence correspondence [7] and this detailed sub-analysis definitely counter this interpretation. A recent systematic review assessing the evidence base for fluid resuscitation in the treatment of children with shock due to sepsis or severe infection found only 13 pediatric trials. Whilst the majority of all randomized evidence to date comes from the FEAST trial, they recommended that implementation of simple algorithms for children managed at hospitals with limited resources to ensure identification of children who maybe potentially be harmed by fluid boluses [35]. The findings we report here raise questions over the rates, volumes and types of solutions recommended in pediatric resuscitation protocols, most of which remain untested in clinical trials.

## Conclusions

The results of the FEAST trial together with findings from these additional analyses suggest that rapid administration of fluid boluses increase the risk of subsequent cardiovascular collapse in children with shock, rather than increasing the risk of fatal fluid overload. The results suggest the need for further research to better understand the pathophysiology of shock and its treatment, and the mechanisms whereby rapid fluid resuscitation increased mortality in African children.

## Abbreviations

CI: confidence interval; ERC: Endpoint Review Committee; FEAST: Fluid Expansion as Supportive Therapy; PS: presentation syndromes; RR: relative risk; SBP: systolic blood pressure; TCE: terminal clinical events.

## Competing interests

The authors declare that they have no competing interests.

## Authors' contributions

KM conceived the trial and participated in the design of the sub-study and wrote the first draft of the manuscript; ECG participated in the design of the study and performed the statistical analysis; JAE chaired the ERC, and participated in the design of the study and the writing of the manuscript; SK coordinated the clinical trial within Uganda, and participated in the design of the sub-study and writing of the manuscript; PO was a member of ERC, and participated in the design of the study and writing of the manuscript; SOA was a member of the ERC, and participated the design of the study and writing of the manuscript; ROO was a member of the ERC, and participated in the data collection and interpretation of data; CE participated in the coordination of the study, was a member of the ERC, and participated in the data collection and interpretation of data; RN participated in the coordination of the study and was a member of the ERC, and participated in the data collection and interpretation of data; GM participated in the coordination of the study, was a member of the ERC, and participated in the data collection and interpretation of data; HR participated in the design of the study and writing of the manuscript; BB carried out the independent review of serious adverse events and was involved in the writing of the manuscript; JN was involved in the coordination of data collection and interpretation; AM carried out the independent review of serious adverse events and was involved in the writing of the manuscript; NP participated in the training of study clinicians and coordination of data collection and data interpretation; CMD participated in the data collection and interpretation of data; DS participated in the data collection and interpretation of data; AD participated in the data collection and interpretation of data; VO participated in the data collection and interpretation of data; RW participated in the data collection and interpretation of data; MT participated in the training of study clinicians, data collection and interpretation of data; BO participated in the data collection and interpretation of data; ML participated the design of the study and writing of the manuscript; JC participated in the design of the study, the ERC and writing of the manuscript, AGB participated in the design of the study and performed the statistical analysis, DMG participated in the design of the study, the ERC and writing of the manuscript. All authors read and approved the final manuscript.

## Pre-publication history

The pre-publication history for this paper can be accessed here:

http://www.biomedcentral.com/1741-7015/11/68/prepub

## Supplementary Material

Additional file 1 **Figure S1: Box and whisker plots and bar charts of bedside vital status observations, oxygen saturation and hypoglycemia by arm (fluid arms combined) versus control arm**. **Table S1**: Admission presentation syndromes by randomization arm (FEAST A only). **Figure S2 **: Hazard ratios and 95% confidence intervals of boluses compared to no bolus for mortality over different levels of oxygen saturation at baseline. **Figure S3**: Hazard ratios and 95% confidence intervals for boluses compared to no bolus for mortality over different levels of hemoglobin at baseline. **Table S2**: Terminal clinical events for 297 children that died within 48 hours by baseline presentation. **Figure S4a**: Cumulative incidence for bolus and no-bolus arms by cardiogenic only, neurological only or respiratory only terminal clinical events. **Figure S4b**: Cumulative incidence by bolus versus no bolus for combined causes: respiratory and neurological, cardiogenic and neurological, and unknown or other terminal clinical events. **Table S3a**: Terminal clinical event by 48 hours by randomization arm (bolus versus no bolus) and by whether they were enrolled before or after the protocol amendment. **Table S3b**: Terminal clinical event by 48 hours by randomization arm (bolus versus no bolus) by malaria status. **Table S3c**: Terminal clinical events in those with anemia and those without anemia at baseline. Percentages are out of all those enrolled in that arm within the anemia group or non-anemia group (totals are at the top of each column). **Table S4a**: Mortality at 48 hours with and without persisting features of shock at one hour. **Table S4b**: Mortality at 48 hours in those with or without hypoxia at baseline. Click here for file
