# Supplementary material for: Exploring mechanisms of excess mortality with early fluid resuscitation: insights from the FEAST trial
Source: BMC Med. 2013 Mar 14;11:68. doi: 10.1186/1741-7015-11-68 (PMC3599745; doi:10.1186/1741-7015-11-68)
Supplement: Additional file 1 — Figure S1: Box and whisker plots and bar charts of bedside vital status observations, oxygen saturation and hypoglycemia by arm (fluid arms combined) versus control arm. Table S1: Admission presentation syndromes by randomization arm (FEAST A only). Figure S2 : Hazard ratios and 95% confidence intervals of boluses compared to no bolus for mortality over different levels of oxygen saturation at baseline. Figure S3: Hazard ratios and 95% confidence intervals for boluses compared to no bolus for mortality over different levels of hemoglobin at baseline. Table S2: Terminal clinical events for 297 children that died within 48 hours by baseline presentation. Figure S4a: Cumulative incidence for bolus and no-bolus arms by cardiogenic only, neurological only or respiratory only terminal clinical events. Figure S4b: Cumulative incidence by bolus versus no bolus for combined causes: respiratory and neurological, cardiogenic and neurological, and unknown or other terminal clinical events. Table S3a: Terminal clinical event by 48 hours by randomization arm (bolus versus no bolus) and by whether they were enrolled before or after the protocol amendment. Table S3b: Terminal clinical event by 48 hours by randomization arm (bolus versus no bolus) by malaria status. Table S3c: Terminal clinical events in those with anemia and those without anemia at baseline. Percentages are out of all those enrolled in that arm within the anemia group or non-anemia group (totals are at the top of each column). Table S4a: Mortality at 48 hours with and without persisting features of shock at one hour. Table S4b: Mortality at 48 hours in those with or without hypoxia at baseline. [file 1741-7015-11-68-S1.DOC]

**Figure S1 Box and whisker plots of bedside observations by fluid arm**

|  |  |
| --- | --- |
|  |  |
|  |  |
|  |  |

**Table S1:** Admission Presentation Syndromes by randomization arm.

| **Presentation Syndrome at baseline** | **Fluid Arm** | | | |  |
| --- | --- | --- | --- | --- | --- |
|  | Albumin | Saline | Combined bolus | No bolus | Total |
|  | N=1050 | N=1047 | N=2097 | N=1044 | N=3141 |
| Acid-base or Lactate measurements available (Classifiable severe shock) | 785 (75%) | 804 (77%) | 1589 (76%) | 807 (77%) | 2396 (76%) |
| *Respiratory only* | 34 (3%) | 31 (3%) | 69 (3%) | 44 (4%) | 109 (3%) |
| *Severe shock/acidosis only* | 218 (21%) | 245 (23%) | 446 (21%) | 230 (22%) | 693 (22%) |
| *Neurological only* | 71 (7%) | 66 (6%) | 146 (7%) | 84 (8%) | 221 (7%) |
| *Respiratory and severe shock* | 86 (8%) | 85 (8%) | 166 (8%) | 84 (8%) | 255 (8%) |
| *Respiratory and neurological* | 16 (2%) | 23 (2%) | 41 (2%) | 17 (2%) | 56 (2%) |
| *Severe shock and neurological* | 171 (16%) | 164 (16%) | 321 (15%) | 159 (15%) | 494 (16%) |
| *All three presentations* | 69 (7%) | 70 (7%) | 134 (6%) | 66 (6%) | 205 (7%) |
| *None of the presentations* | 120 (11%) | 120 (11%) | 257 (12%) | 123 (12%) | 363 (12%) |
|  |  |  |  |  |  |
| Non-ascertainable severe shock/acidosis status* | 227 (22%) | 202 (19%) | 429 (20%) | 204 (20%) | 633 (20%) |
| *- with respiratory* | *27 (3%)* | *24 (2%)* | *51 (2%)* | *30 (3%)* | *81 (3%)* |
| *- with neurological* | *75 (7%)* | *61 (6%)* | *136 (6%)* | *46 (4%)* | *182 (6%)* |
| *- with respiratory and neurological* | *16 (2%)* | *10 (1%)* | *26 (1%)* | *19 (2%)* | *45 (1%)* |
| *- with neither respiratory nor neurological* | *109 (11%)* | *107 (10%)* | *216 (10%)* | *109 (11%)* | *325 (10%)* |
|  |  |  |  |  |  |
| Missing information on two or more presentations | 38 (4%) | 41 (4%) | 79 (4%) | 33 (3%) | 112 (4%) |

NB Percentages are out of all those enrolled into that randomization arm

*see definition of severe shock; children with lactic acid or base deficit or systolic blood pressure values missing were categorized as having ‘non-ascertainable severe shock status’.

**Figure S2 : 48-hour mortality by baseline oxygen saturation level; hazard ratio for bolus versus no bolus.**

**Figure S3: 48-hour mortality by baseline haemoglobin level; hazard ratio for bolus versus no bolus.**

**Table S2: Terminal Clinical Events in children that died within 48 hours by baseline presentation.**

| Presentation at baseline | Terminal Clinical Event * | | | | | | |  |
| --- | --- | --- | --- | --- | --- | --- | --- | --- |
|  | Respiratory only | Cardiogenic only | Neurological only | Respiratory + Cardiogenic | Respiratory  +  Neurological | Cardiogenic  +  Neurological | Unknown | Total |
| Respiratory only | 3 (100%) | 0 (0%) | 0 (0%) | 0 (0%) | 0 (0%) | 0 (0%) | 0 (0%) | 3 (100%) |
| Severe shock/acidosis only | 11 (20%) | 34 (61%) | 5 (2%) | 0 (0%) | 1 (2%) | 1 (2%) | 4 (7%) | 56 (100%) |
| Neurological only | 1 (20%) | 0 (0%) | 3 (60%) | 0 (0%) | 0 (0%) | 1 (20%) | 0 (0%) | 5 (100%) |
| Respiratory + severe shock | 21 (45%) | 22 (47%) | 1 (2%) | 0 (0%) | 1 (2%) | 0 (0%) | 2 (4%) | 47 (100%) |
| Respiratory + neurological | 1 (50%) | 0 (0%) | 0 (0%) | 0 (0%) | 1 (50%) | 0 (0%) | 0 (0%) | 2 (100%) |
| Severe shock +neurological | 5 (7%) | 21 (29%) | 33 (45%) | 0 (0%) | 7 (9%) | 4 (5%) | 4 (5%) | 74 (100%) |
| All three presentations | 7 (13%) | 19 (36%) | 15 (28%) | 0 (0%) | 7 (14%) | 3 (6%) | 2 (4%) | 53 (100%) |
| None of the presentations | 0 (0%) | 2 (67%) | 1 (33%) | 0 (0%) | 0 (0%) | 0 (0%) | 0 (0%) | 3 (100%) |
| Unascertainable shock/acidosis status with either respiratory or neurological presentations | 8 (38%) | 3 (14%) | 2 (10%) | 0 (0%) | 2 (10%) | 1 (5%) | 5 (24%) | 21 (100%) |
| - respiratory only | 3 (60%) | 1 (20%) | 0 (0%) | 0 (0%) | 0 (0%) | 0 (0%) | 1 (20%) | 5 (100%) |
| - neurological only | 1 (14%) | 1 (14%) | 2 (29%) | 0 (0%) | 0 (0%) | 0 (0%) | 3 (43%) | 7 (100%) |
| - respiratory + neurological | 1 (33%) | 0 (0%) | 0 (0%) | 0 (0%) | 1 (33%) | 0 (0%) | 1 (33%) | 3 (100%) |
| - neither respiratory nor neurological | 3 (50%) | 1 (17%) | 0 (0%) | 0 (0%) | 1 (17%) | 1 (17%) | 0 (0%) | 6 (100%) |
| Missing information on two or more presentations | 4 (12%) | 22 (67%) | 3 (9%) | 0 (0%) | 2 (6%) | 1 (3%) | 1 (3%) | 33 (100%) |
| Total | 61 (21%) | 123 (41%) | 63 (21%) | 0 (0%) | 21 (7%) | 11 (4%) | 18 (6%) | 297 (100%) |

* percentages are for each row and indicate how many of the deaths in each baseline presentation group go on to die of each terminal clinical event.

**Figure S4a: Cumulative incidence of cardiogenic only, neurological only or respiratory only TCE**

**Figure S4b: Cumulative incidence for combined causes: respiratory and neurological, cardiogenic and neurological, and unknown/other TCE**

**Table S3a: TCE by 48 hours by randomisation arm before or after the protocol amendment.**

|  | Before/after protocol amendment and randomisation arm (n=3141) | | | | | | | |
| --- | --- | --- | --- | --- | --- | --- | --- | --- |
| Terminal Clinical Event | Before amendment | | | | After amendment | | | |
|  | Combined bolus | No bolus | Total | Subhazard ratio | Combined bolus | No Bolus | Total | Subhazard ratio |
| Total enrolled | 1691 | 844 | 2535 |  | 406 | 200 | 606 |  |
| Cardiogenic (ongoing signs of shock) only | 73 (4.3%) | 23 (2.7%) | 96 (3.8%) | 1.59  (1.00-2.55) | 23 (5.7%) | 4 (2%) | 27 (4.5%) | 2.88  (0.99-8.37) |
| Neurological only | 33 (1.9%) | 15 (1.8%) | 48 (1.9%) | 1.10  (0.60-2.02) | 11 (2.7%) | 4 (2%) | 15 (2.5%) | 1.36  (0.43-4.28) |
| Respiratory only | 37 (2.2%) | 11 (1.3%) | 48 (1.9%) | 1.69  (0.86-3.31) | 10 (2.5%) | 3 (1.5%) | 13 (2.1%) | 1.66  (0.46-6.03) |
| Cardiogenic and Neurological | 7 (0.4%) | 0 (0%) | 7 (0.3%) | NA | 3 (0.7%) | 1 (0.5%) | 4 (0.7%) | 1.48  (0.15-14.3) |
| Respiratory and Neurological | 11 (0.7%) | 8 (0.9%) | 19 (0.7%) | 0.69  (0.28-1.70) | 0 (0%) | 2 (1%) | 2 (0.3%) | NA |
| Unknown/Other | 11 (0.7%) | 5 (0.6%) | 16 (0.6%) | 1.10  (0.38-3.16) | 2 (0.5%) | 0 (0.0%) | 2 (0.3%) | NA |

NB: Percentages are out of all those enrolled in that arm either before or after the protocol amendment (totals are at the top of each column).

**Table S3b: Terminal Clinical Event by 48 hours by randomisation arm by malaria status.**

|  | Malaria status and randomisation arm (n=3123*) | | | | | | | |
| --- | --- | --- | --- | --- | --- | --- | --- | --- |
| Terminal Clinical Event | Malaria | | | | Non-malaria | | | |
|  | Combined bolus | No bolus | Total | Subhazard ratio | Combined bolus | No Bolus | Total | Subhazard ratio |
| Total enrolled | 1202 | 591 | 1793 |  | 884 | 446 | 1330 |  |
| Cardiogenic (ongoing signs of shock) only | 58 (4.8%) | 14 (2.4%) | 72 (4.0%) | 2.06  (1.15-3.70) | 35 (3.9%) | 12 (2.7%) | 47 (3.5%) | 1.48  (0.77-2.85) |
| Neurological only | 24 (2.0%) | 9 (1.5%) | 33 (1.8%) | 1.31  (0.61-2.83) | 20 (2.3%) | 9 (2.0%) | 29 (2.2%) | 1.12  (0.51-2.46) |
| Respiratory only | 13 (1.1%) | 5 (0.8%) | 18 (1.0%) | 1.28  (0.46-3.59) | 34 (3.8%) | 9 (2.0%) | 43 (3.2%) | 1.94  (0.93-4.02) |
| Cardiogenic and Neurological | 5 (0.4%) | 0 (0.0%) | 5 (0.3%) | NA | 5 (0.6%) | 1 (0.2%) | 6 (0.5%) | 2.52  (0.29-21.67) |
| Respiratory and Neurological | 6 (0.5%) | 6 (1.0%) | 12 (0.7%) | 0.49  (0.16-1.52) | 5 (0.6%) | 4 (0.9%) | 9 (0.7%) | 0.63  (0.17-2.34) |
| Unknown/Other | 4 (0.3%) | 0 (0%) | 4 (0.2%) | NA | 9 (1.0%) | 3 (0.7%) | 12 (0.9%) | 1.51  (0.41-5.60) |

* There are 18 children that have a missing malaria result.

NB: Percentages are out of all those enrolled in that arm within the malaria group or non-malaria group (totals are at the top of each column)

**Table S3c: Terminal Clinical Events in those with anemia and those without anemia at baseline.**

|  | Severe Anemia (<5g/dl haemoglobin) or no anemia (≥5g/dl haemoglobin) (n=3054*) | | | | | | | |
| --- | --- | --- | --- | --- | --- | --- | --- | --- |
| Terminal Clinical Event /Syndrome | Severe Anemia | | | | Haemoglobin ≥ 5g/dl | | | |
|  | Combined bolus | No bolus | Total | Subhazard ratio° | Combined bolus | No Bolus | Total | Subhazard ratio° |
| Total enrolled | 655 | 332 | 987 |  | 1384 | 683 | 2067 |  |
| Cardiogenic (ongoing signs of shock) only | 69 (11%) | 19 (6%) | 88 (9%) | 1.87  (1.12-3.12) | 23 (2%) | 7 (1%) | 30 (1%) | 1.63  (0.70-3.79) |
| Neurological only | 7 (1%) | 5 (2%) | 12 (1%) | 0.71  (0.23-2.23) | 37 (3%) | 14 (2%) | 51 (2%) | 1.3  (0.70-2.41) |
| Respiratory only | 12 (2%) | 1 (0.3%) | 13 (1%) | 6.12  (0.79-47.3) | 34 (2%) | 13 (2%) | 47 (2%) | 1.3  (0.69-2.46) |
| Cardiogenic and Neurological | 6 (1%) | 1 (0.3%) | 7 (0.7%) | 3.04  (0.36-25.4) | 4 (0.3%) | 0 (0%) | 2 (0.2%) | NA |
| Respiratory and Neurological | 2 (0.3%) | 3 (1%) | 5 (0.5%) | 0.34  (0.05-2.01) | 8 (0.6%) | 6 (0.9%) | 14 (0.7%) | 0.66  (0.23-1.89) |
| Unknown/Other | 5 (1%) | 1 (0.3%) | 6 (0.6%) | 2.54  (0.30-21.7) | 8 (0.6%) | 3 (0.4%) | 11 (0.5%) | 1.32  (0.35-4.96) |

*There are 87 children without a haemoglobin result at baseline.

°The sub-hazard ratio for bolus combined vs. no bolus takes into account the competing risks.

**Table S4a: Mortality at 48 hours children with and without persisting features of shock at one hour**

|  | Albumin | Saline | Bolus combined | No Bolus | Total^¥^ | Relative risk* |
| --- | --- | --- | --- | --- | --- | --- |
| No shock at one hour | 21/445 (5%) | 22/430 (5%) | 43/876  (5%) | 8/323  (2%) | 51/1198  (4%) | 1.98  (0.94-4.17) |
| Continued shock at one hour | 71/584 (12%) | 70/596 (12%) | 141/1180 (12%) | 50/701  (7%) | 191/1881 (10%) | 1.67  (1.23-2.28) |

*p-value for heterogeneity between the two relative risks. = 0.68

^¥^Measurements available for 3079/3141 children as 39 had died and 23 had missing impaired perfusion at one hour.

**Table S4b: Mortality at 48 hours in those with or without hypoxia by their baseline status.**

|  | Hypoxic at baseline | | | | |  |
| --- | --- | --- | --- | --- | --- | --- |
|  | Albumin | Saline | Bolus | No Bolus | Total^¥^ | Relative Risk |
| No hypoxia at one hour | 9/165 (5%) | 17/172 (10%) | 26/337 (8%) | 11/205 (5%) | 37/542 (7%) | 1.44  (0.73-2.85)* |
| Continued hypoxia at one hour | 17/101 (17%) | 14/90 (16%) | 31/191 (16%) | 19/83 (23%) | 50/274 (18%) | 0.71  (0.43-1.18)* |
|  | Without hypoxia at baseline | | | | |  |
| No hypoxia at one hour | 44/661 (7%) | 35/651 (5%) | 79/1312 (6%) | 15/657 (2%) | 94/1969 (5%) | 2.64  (1.53-4.54)^†^ |
| Became hypoxic at one hour | 10/62 (16%) | 12/67 (18%) | 22/129 (17%) | 4/46 (9%) | 26/175 (15%) | 1.96  (0.71-5.39) ^†^ |

*p-value for heterogeneity between risk ratios for those hypoxic at baseline p=0.10.

^†^p-value or heterogeneity between risk ratios for those without hypoxia at baseline p=0.63

^¥^Measurements available for 2960/3141 children as 39 had died and 142 had missing oxygen saturation (103 at baseline, 39 at one hour.
